# Adrenal Vein Sampling in the Management of Primary Aldosteronism: The Added Value of Intraprocedural Cortisol Assessment

**DOI:** 10.1155/2023/5563881

**Published:** 2023-12-21

**Authors:** Inês Manique, Sara Amaral, Alexandra Matias, Bruno Bouça, Salomé Serranito, João Torres, Olga Gutu, Tiago Bilhim, Élia Coimbra, Isaura Rodrigues, Conceição Godinho, Luísa Cortez, José Silva-Nunes

**Affiliations:** ^1^Department of Endocrinology, Diabetes and Metabolism of Centro Hospitalar Universitário Lisboa Central, Hospital de Curry Cabral, Lisbon, Portugal; ^2^Nova Medical School, Faculdade de Ciências Médicas, Universidade Nova de Lisboa, Lisbon, Portugal; ^3^Department of Interventional Radiology of Centro Hospitalar Universitário Lisboa Central, Hospital de Curry Cabral, Lisbon, Portugal; ^4^Department of Laboratory Medicine, Clinical Pathology of Centro Hospitalar de Lisboa Central, Lisboa, Portugal; ^5^Health and Technology Research Center (H&TRC), Escola Superior de Tecnologia da Saúde de Lisboa, Lisbon, Portugal

## Abstract

**Introduction:**

Primary aldosteronism is the most common cause of secondary hypertension. Adrenal vein sampling is the gold standard for subtyping primary aldosteronism. However, this procedure is technically challenging and often has a low success rate. Our center is one of the very few performing this technique in our country with an increasing experience.

**Objective:**

The aim of this study was to evaluate the role of the cortisol intraprocedural assay in improving the performance of adrenal vein sampling.

**Design:**

We enrolled all of the patients with primary aldosteronism that underwent adrenal vein sampling from February 2016 to April 2023. The cortisol intraprocedural assay was introduced in October 2021.

**Methods:**

We enrolled a total of 50 adrenal vein samplings performed on 43 patients with the diagnosis of primary aldosteronism. In this sample, 19 patients and 24 patients underwent adrenal vein sampling before and after intraprocedural cortisol measurement, respectively. The procedure was repeated in seven patients (one before and six after intraprocedural cortisol measurement), given the unsuccess of the first exam. Selectivity of the adrenal vein sampling was assumed if the serum cortisol concentration from the adrenal vein was at least five times higher than that of the inferior vena cava. Lateralization was assumed if the aldosterone to cortisol ratio of one adrenal vein was at least four times the aldosterone to cortisol ratio of the contralateral side.

**Results:**

The mean age of the patients that underwent adrenal vein sampling (*N* = 43) was 55.2 ± 8.9 years, and 53.5% (*n* = 23) were female. The mean interval between the diagnosis of hypertension and the diagnosis of primary aldosteronism was 9.8 years (±9.9). At diagnosis, 62.8% of the patients (*n* = 27) had hypokalemia (mean value of 3 mmol/L (±0.34)), 88.4% (*n* = 38) had adrenal abnormalities on preprocedural CT scan, and 67.4% (*n* = 29) described as unilateral nodules. There were no statistically significant differences in the patients' baseline characteristics between the two groups (before and after intraprocedural cortisol measurement). Before intraprocedural cortisol measurement, adrenal vein sampling selectivity was achieved in 35% (*n* = 7) patients. Selectivity increased to 100% (30/30) after intraprocedural cortisol measurement (*p* < 0.001). With the exception of one patient who refused it, all patients with lateralized disease underwent unilateral adrenalectomy with normalization of the aldosterone to renin ratio postoperatively.

**Conclusions:**

The lack of effective alternatives in subtyping primary aldosteronism highlights the need to improve the success rate of adrenal vein sampling. In this study, intraprocedural cortisol measurement allowed a selectivity of 100%. Its addition to this procedure protocol should be considered, especially in centers with a low success rate.

## 1. Introduction

Primary aldosteronism (PA) affects an estimated 6% of all patients with hypertension and 20% of those with resistant hypertension, being the most common cause of secondary hypertension [1]. In PA, there is an autonomous production of aldosterone with low renin levels [1]. Aldosterone acts in the principal cells through the activation of the mineralocorticoid receptor of the distal tubule and collecting duct, mediating sodium resorption and secretion of potassium and hydrogen. Reabsorption of sodium is one of the main effects associated with hypertension in PA. Other effects of aldosterone include blood vessel remodeling, fibrosis and endothelial dysfunction, cardiac fibrosis and hypertrophy, and atrial fibrillation [2]. PA may be unilateral, usually caused by an aldosterone-producing adenoma, or bilateral caused by bilateral adrenal hyperplasia. The two main strategies to treat PA include unilateral adrenalectomy or lifelong medical therapy with mineralocorticoid receptor antagonists (MRAs). Most studies show that surgical treatment leads to more favorable results than medical therapy. Patients on MRA maintain residual disease, as suggested by higher blood pressure values, lower serum potassium, higher rates of atrial fibrillation, a decline of renal function, decreased quality of life (QoL), and increased mortality. This may be attributable to insufficient dosing of medical treatment in the setting of a lack of adherence to prescribed MRA therapy and/or an insufficient uptitration of MRA therapy. Another explanation for the better outcomes of surgical therapy could be related to the possible concomitant cortisol cosecretion that is corrected by adrenalectomy but not by MRA treatment. In fact, up to 78% of PA patients exhibit at least one pathological test result for excess glucocorticoid [3, 4].

To define the best treatment strategy, subtyping PA is crucial and adrenal vein sampling (AVS) is the gold standard test. However, this procedure is challenging and expensive [5]. Success rates for the AVS procedure range from 40 to 94%. The anatomical complexity and variations lead to frequent AVS failures [6]. Cannulation of the right adrenal vein is the most challenging part of AVS, which is also the most common cause of unsuccessful AVS [7]. The confirmation of the technical success of the procedure is often not available until hours or days after its performance, usually after the patient has already been discharged, making resampling cumbersome and bearing higher costs [8]. In 2007, the first proof of concept study was published by an Italian group showing the feasibility of rapid on-site measurement of cortisol during the AVS study in five subjects. Since then, other studies have been published demonstrating the improvement of successful AVS when using intraprocedural cortisol assays [8, 9].

The purpose of this study is to report and review the role of the cortisol intraprocedural assay in the sampling of adrenal veins in PA in our center.

## 2. Methods

### 2.1. Patients

We enrolled 43 patients with the diagnosis of PA who underwent the AVS procedure from February 2016 to April 2023. This corresponded to 19 patients and 24 patients that performed AVS before and after intraprocedural cortisol measurement (IPCM) introduction, respectively.

Data were collected on the patient's characteristics, i.e., sex, age at diagnosis of hypertension diagnosis and age at diagnosis of PA, complications possibly associated with hypertension, antihypertensive drugs, serum potassium at diagnosis of PA, and findings on adrenal computed tomography (CT).

Given the possible interference with AVS results, all patients stopped MRA at least six weeks and diuretics four weeks before the procedure. Alternative antihypertensive drugs used included nifedipine, doxazosin, and diltiazem.

This retrospective study was approved by the Ethics Committee of the Centro Hospitalar Universitário Lisboa Central. All patients had given their written consent for the AVS procedure.

### 2.2. CT Technique

All patients had adrenal CT before AVS for mapping of the adrenal veins and diagnosis. In addition to axial (3 mm slice thickness), multiplanar sagittal and coronal reformations (3 mm slice thickness) were used.

### 2.3. AVS

AVS was performed by a team of two interventional radiologists. Before the procedure, patients' blood was collected for analysis and normal serum potassium was ensured before AVS (potassium ≥3.5 mmol/L).

All the patients followed the protocol of our center with ACTH stimulation. ACTH perfusion was started 30 min before the procedure. All procedures were performed in fasting to reduce the effect of food intake in aldosterone levels and in the morning given the circadian fluctuations in aldosterone levels. They started at 8 am and took about two hours. The procedures encompassed the operator learning curves for both interventional radiologists who had no prior experience with these procedures and did not receive proctoring. Both interventional radiologists had experience in vascular and catheter-based procedures for 25 and 10 years before the start of this study. All procedures were performed from a femoral vein access (5Fr). For the left adrenal vein, a 4Fr Berenstein catheter was used (Cordis), and for the right adrenal vein, a 5Fr Cobra 1 or Simmons 1 catheter was used (Cordis). All catheters were used on 0.035-inch hydrophilic guidewires (Terumo). Side holes were created near the catheter tip with the use of an intramuscular injection needle.

Blood was collected in EDTA K3E tubes (Sarstedt). The team started performing the cortisol intraprocedural assay in October 2021. The radiologist started to collect blood in the right adrenal vein. The test tubes were immediately transferred to the endocrinology laboratory, centrifuged for five minutes at 3000 g, and the supernatant plasma was used for immediate cortisol analysis. The time for transport, centrifugation, and measurement was 60–120 min, during which the radiologist attempted sampling of the contralateral adrenal vein. The sampling catheter was not left in position in the adrenal vein during the measurement. The patient stayed in the radiology suite until the last sample was analyzed. Following the procedure, the patient remained in the day case ward for four hours.

### 2.4. Hormone Assays

Cortisol concentrations were determined using an automated chemiluminescence microparticle immunoassay (ARCHITECT Abbott), and aldosterone concentrations were also measured using an automated chemiluminescence microparticle immunoassay (LIAISON Diasorin®). In August 2022, the determination of cortisol started to be carried out on Roche equipment Cobas e601, an automated eletrochemiluminescence microparticle immunoassay. Cortisol's technical execution time was 40 minutes but with the use of the new referred equipment, it substantially reduced the technical execution time in the equipment (from 40 minutes to 18 minutes). The samples with cortisol values above 59.8 *μ*g/dL had serial dilutions (1/10 or 1/20 or 1/40 until the final value). The interval for the measurement of aldosterone samples was 0.97–100 ng/dL. Values above 100 ng/dL had serial dilutions until the final value. Cortisol was reported in *μ*g/dl and aldosterone in ng/dl.

### 2.5. Selectivity and Lateralization Criteria

Selective adrenal sampling was assumed if the cortisol concentration in the adrenal vein was at least five times higher than that in the sample drawn simultaneously from the catheter sheath in the inferior vena cava (IVC) (cortisol in the adrenal vein/cortisol in IVC vein >5.0). Bilateral selectivity was a condition for assuming a successful AVS, as it means that correct cannulation of both the adrenal veins was performed. The diagnosis of unilateral aldosterone secretion was made if the aldosterone to cortisol ratio from one adrenal vein was at least four times the aldosterone to cortisol ratio of the contralateral side. Bilateral aldosterone secretion in the context of a bilateral adrenal hyperplasia was assumed if the ratio of aldosterone to cortisol from one adrenal vein was below three times the ratio of the contralateral side. If this ratio was between three and four, a “grey zone” was considered and no conclusions were made [10].

### 2.6. Procedure Costs

The total cost of each AVS considering the patient hospitalization, ACTH infusion, AVS procedure, and hormonal assays was 4048,51€.

### 2.7. Statistical Analysis

Categorical variables are presented as absolute numbers and percentages. Continuous variables with non-normality distribution are presented as the median ± interquartile range. We used the Mann–Whitney test to compare continuous variables. Categorical variables were compared using *χ*^2^. Data collection and registration were performed with Microsoft Excel and statistical analysis with IBM SPSS Statistics 23rd version for Windows (IBM, Armonk, NY, USA). Statistical significance was determined with a two-sided *p* value <0.05.

## 3. Results

### 3.1. Patients' Characteristics

A total of 43 patients underwent AVS (19 before and 24 after IPCM). The patients' characteristics are reported in Table 1. The mean age was 55.2 ± 8.9 years, and 53.5% (*n* = 23) were female. At the baseline, patients were taking a mean of 2.8 ± 1.1 antihypertensive drugs. The mean age at hypertension diagnosis was 42.3 ± 11.5 years ranging between 15 and 67 years. The mean age at PA diagnosis was 52.1 ± 9.5 years ranging between 31 and 72 years. The mean interval time between hypertension and PA diagnosis was 9.8 ± 9.9 years. Hypokalemia was observed in 27 patients (62.8%) with a mean of 3 mmol/L (±0.34).

Eleven patients (25.6%) had at least one complication attributable to hypertension, the most common being cardiopathy (*n* = 8; 18.6%) and cerebrovascular disease (*n* = 4; 9.3%).

There were no statistically significant differences between the two groups (before and after IPCM), as shown in Table 1.

Globally, 38 patients (88.4%) had adrenal abnormalities on preprocedural CT scan. The majority (*n* = 29; 67.4%) were described as unilateral nodules (16 right-sided and 13 left-sided) ranging from 2.9 mm to 35 mm (mean 17.3 ± 7.4 mm). In patients with bilateral nodes (*n* = 5; 11.6%), the dimension was 18.4 ± 7.1 mm in the right lesions and 15.8 ± 5.8 mm in the left lesions. In three patients, abnormalities were described as unilateral hyperplasia (two left-sided and one right-sided). Both adrenals were considered normal in six (14%) of the patients.

### 3.2. AVS Data

**Figure 1:**
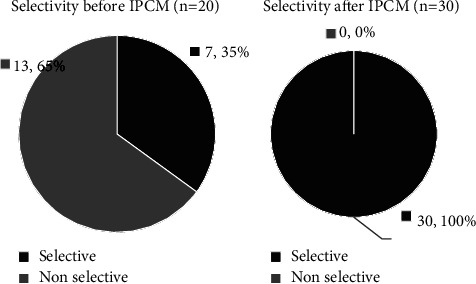
Selectivity in AVS before (2016–September 2021) and after (October 2021–April 2023) intraprocedural cortisol measurement.

A total of 50 AVSs were performed in the considered period of time (20 before and 30 after IPCM), with seven of them corresponding to repetitions (one before and six after IPCM introduction) given the unsuccess of the first procedure. Globally, selectivity was achieved in 37 AVSs (74%). The rate of bilateral selectivity before IPCM (2016–September 2021) was 35% (7/20) and after the cortisol assay (October 2021–April 2023) was 100% (30/30) (*p* < 0.001). The cause of unsuccessful AVS was nonachieving right adrenal selectivity (*n* = 13). In three of them, selectivity was also not achieved in the left adrenal vein samples.  Figure 1 shows the difference in selectivity before and after IPCM.

Among patients with bilaterally successful AVS after IPCM (*n* = 30), the median selective index was 16.7 (12.1–32.3) in the samples of the right adrenal vein and 26.9 (14.9–40.6) in the left adrenal. Among the patients with bilaterally successful AVS before IPCM (*n* = 7), the median selective index was 21.8 (16.4–37.9) in samples from the right adrenal vein samples (*p* = 0.03 vs. after IPCM) and 18.8 (15.5–23) in left adrenal vein samples (*p* = 0.1 vs. after IPCM).

Lateralization was obtained in 46.7% (*n* = 14) of the patients who underwent AVS after IPCM, with a median cortisol-corrected aldosterone lateralization index of 20.8 (12.1–25.9). Lateralization was found in 71.4% (*n* = 5) of the patients who underwent AVS before IPCM, with a median lateralization index of 24.1(12.7–38; *p* = 0.01 vs. after IPCM).

Right-sided lateralization was the most common, occurring in 14 of the 19 patients (73.7%). Right lateralization was verified in four AVS performed before IPCM (80%) and in ten AVS after IPCM (71.4%). Left lateralization occurred in one AVS before IPCM (20%) and in four AVS after IPCM (13.3%) (*p* = 0.36).  Table 2 shows the lateralization ratios considering the different AVS results before and after IPCM.

There were no statistically significant differences between lateralization and the number of antihypertensive medications (*p* = 0.4), presence of hypokalemia (*p* = 0.12), or complications attributable to hypertension (*p* = 0.68).

All AVS procedures occurred without complications. Two patients complained of nausea, sweating, and palpitations after starting the tetracosactide infusion. No major side effects were reported.

Table 3 shows AVS outcomes considering preprocedural imaging and respective outcomes in terms of success of the procedure.

Among all bilaterally selective AVSs (*n* = 37), the discordance between AVS and CT scan was observed in 51.4% of the patients (*n* = 19).

### 3.3. Clinical Outcomes

Except for one patient that refused it, all patients with lateralized AVS (13 in concordance and five nonconcordant to the preprocedure imaging) underwent unilateral adrenalectomy. After surgery, the patients reduced their medication from an average of 2.93 (±1.1) to 0.8 (±1.1) antihypertensive drugs. All the histopathological exams revealed a cortical adenoma, and a cure of PA was verified. Postoperatively, there was a normalization of the aldosterone to renin ratio (aldosterone to renin ratio (ARR) <3.7) with a mean ARR of 1.8 (±0.94).

Patients with bilateral disease (with no lateralization in AVS and with assumed bilateral adrenal hyperplasia) were kept under medical therapy. Patients with “grey zone” AVS results were also maintained under medical therapy. All these patients had controlled blood pressure (<140/90 mmHg in ambulatory report and in the appointment) with an average of 2.6 (±1.3) antihypertensive drugs.

## 4. Discussion

AVS is an essential diagnostic procedure in the workup of PA. Adrenalectomy is the treatment of choice in cases with aldosterone overproduction from one adrenal gland. Rossi et al. and PASO study, a large multicenter study, like other studies, documented the clinical benefits of AVS-guided selection of surgical candidates [11, 12].

The AVS success rate in our center significantly improved after incorporating IPCM into our practice. This AVS protocol was created by a multidisciplinary team including endocrinology, clinical pathology, and interventional radiology specialties. To the best of our knowledge and investigation, this is the first published analysis of AVS results in our country. The Centro Hospitalar Universitário de Lisboa Central (CHULC) is one of the very few centers in Portugal that is performing AVS, and the growing experience is increasing the referral of patients from other hospitals that do not perform this procedure. The AVS is being performed by the same interventional radiology team.

The differentiation between unilateral and bilateral PA should be based on the results of AVS as recommended by the Endocrine Society guidelines concerning the management of PA [10]. In fact, the CT/MRI imaging findings may be misleading [12, 13]. Discordance rates between imaging and functional studies using AVS are typically 30–40% [14]. In our investigation, in 51.4% of the patients, there was no concordance between imaging and AVS results.

Despite being the gold standard, AVS is challenging and frequently unsuccessful, requiring higher experience. Nuclear medicine studies using positron-emitting agents such as [11C]-metomidate or [68 Ga]-pentixafor can detect small tumors, but the high cost and limited availability give little advantage over AVS, at least currently [8, 12, 15].

The infusion of cosyntropin may increase the accuracy of AVS, increasing the selective index and thus the confidence of correct catheter placement [14]. Its use can also decrease potential ACTH-dependent hormonal fluctuations [12]. In this study, all the patients underwent stimulated AVS with cosyntropin according to our protocol.

Adrenal vein cannulation success is determined by a higher cortisol concentration in the adrenal vein sample than in a paired peripheral plasma sample (selectivity ratio). However, this finding is usually only confirmed after the conclusion of the procedure and after the discharge of the patient [13]. In our study, we considered the cutoff suggested by the guidelines of the Endocrine Society (2016) for the ACTH-stimulated AVS [10]. Some studies such as Mulatero et al. showed that too permissive criteria for successful catheterization led to poor diagnostic reproducibility [16].

The low success rate of AVS (35%) in our center urged the need to optimize the procedure to achieve better results. In September 2021, we reformulated our AVS protocol to include IPCM. During the cortisol assay total run time, the radiologist attempted sampling the contralateral adrenal of the same patient.

IPCM has been found to increase the success rate of AVS, allowing a rapid feedback on the selectivity index and, therefore, the success of the procedure. In cases of nonselectivity, this approach allows the resampling while the patient is still in the procedural room. It does not increase discomfort for the patient and adds only the expense of additional procedural time. Furthermore, IPCM had the advantage of not requiring the use of equipment other than that already existing in the hospital laboratory, with no investment costs associated [8].

In Auchus et al.'s study, the success rate increased from 73% to 97% after the use of cortisol assay during AVS. In this study, the AVS was performed during a continuous infusion of cosyntropin and AVS samples were assayed using an immunochemiluminometric assay. The total turnaround time for serum cortisol assays averaged one to two hours. No adverse effects occurred and the procedure was done by five different interventional radiologists [17]. Rossi et al. showed a final rate of bilateral selectivity higher than historical series (23/25 vs 16/25) with rapid cortisol measurement during AVS. As in this study, our samples were tested as such and after serial dilution [15].

Betz et al. suggested that the rapid cortisol measurement favors better AVS outcomes, mainly in centers with low success rates, due to an initial effect by resampling of the right adrenal vein and a more delayed training effect [8]. Given that, in that study, the success rate previous to the rapid cortisol measurement was low (55%) [8]. Similarly, in our study, before IPCM, our AVS success rate was only 35%.

The increased bilateral selectivity in AVS may also be a consequence of increased experience of interventional radiologist. The AVS learning curve is estimated to be about 20–30 procedures, with maintenance of proficiency of about 15 annual cases [15]. In our study, it probably contributed to the differences in technical success observed before and after the implementation of IPCM during AVS.

The prevalence of unilateral subtypes of PA is comparable to other published studies. There is evidence suggesting a worse clinical prognosis in unilateral disease [7]. In our study, there were no statistically significant differences between lateralization and the number of antihypertensive medications, presence of hypokalemia, or complications of PA. However, we must take into account that the small number of patients enrolled in this study, namely, with unilateral disease, may compromise the detection of statistical differences.

The prevalence of hypokalemia (62.8%) was higher in our study than typically described in the literature. Nevertheless, there are some comparable studies that had similar percentages as that by Rossi et al. (70% of the patients with hypokalemia) [18]. This higher prevalence may reveal a selection bias. Hypokalemia with hypertension is a common reason to investigate a possible PA and, therefore, is responsible for some of the referrals for performing AVS [15].

Limitations to this study beyond the ones already discussed include the retrospective design and nonrandomization of the compared cohorts. The low number of AVS performed can compromise the statistical power of the study. However, the results reinforce the existing evidence, highlighting the importance of the AVS and especially of the IPCM. There is still no universal consensus AVS protocol, namely, in terms of the use of ACTH and the selective and lateralization indices.

Ideally, the determination of intraprocedural cortisol assay should be performed using a point of care testing during the AVS in order to minimize the time elapsed between right adrenal vein sampling and confirmation of a successful catheterization. However, this portable measurement system is not available at the time and, if it existed, it would require a study to assess the accuracy and reproducibility of cortisol compared to the one used by the clinical pathology laboratory in order to validate its use. From September 2022, the determination of cortisol performed on Roche equipment Cobas e601 Roche substantially reduced the technical execution time (from 40 minutes to 18 minutes), consequently reducing the total response time (in about 30 minutes) for confirmation of the technical success of the AVS study.

## 5. Conclusions

In conclusion, AVS remains the gold standard for differentiating aldosterone-producing adenoma from bilateral adrenal hyperplasia. Cannulation of the right adrenal vein remains the major technical challenge in this procedure. IPCM may be an effective tool to improve the AVS success rate, especially in centers with a low success rate.

## Figures and Tables

**Table 1:** Characteristics of patients submitted to adrenal vein sampling.

	Preintraprocedural cortisol measurement (*n* = 19) 2016–September 2021	Postintraprocedural cortisol measurement (*n* = 24) October 2021–December 2022	*P* value	Total (*n* = 43)
Age (years)			0.45	
Mean (SD)	56.4 (±8.2)	54.3 (±9.4)		55.2 (±8.9)
Minimum	40	32		32
Maximum	73	73		73
Gender			0.60	
Female, *n* (%)	11 (57.9%)	12 (42.1%)		23 (53.5%)
Age at hypertension diagnosis			0.59	
Mean (SD)	41.2 (±10.6)	43.1 (±12.3)		42.3 (±11.5)
Minimum	21	34		15
Maximum	60	52		67
Age at PA diagnosis			0.48	
Mean (SD)	53. (±9.6)	51.2 (±9.5)		52.1 (±9.5)
Minimum	34	49		31
Maximum	72	70		72
The interval between hypertension and PA diagnosis (years)			0.20	
Mean	12.1 (±10.6)	8.0 (±9.1)		9.8 (±9.9)
Minimum	2	0		0
Maximum	41	25		41
Number of antihypertensive medication			0.06	
Mean (SD)	3.2 (±0.98)	2.5 (±1.2)		2.8 (±1.1)
Minimum	1	1		1
Maximum	4	5		5
Complications	7 (36.8%)	4 (16.7%)	0.13	11 (25.6%)
Retinopathy	1 (5.3%)	1 (4.2%)	0.87	2 (4.7%)
Cardiopathy	5 (26.3%)	3 (12.5%)	0.13	8 (18.6%)
Nephropathy	0 (0%)	2 (8.3%)	0.20	2 (4.7%)
Cerebrovascular	3 (15.8%)	1 (4.2%)	0.19	4 (9.3%)
Encephalopathy	0 (0%)	1 (4.2%)	0.37	1 (2.3%)
Hypokalemia	13 (68.4%)	14 (58.3%)	0.50	27 (62.8%)
Mean serum K^+^ (mmoL/L) (SD)	3.0 (±0.4)	3.0 (±0.2)		3 (±0.34)
Imaging (CT)			0.95	
Normal adrenals	3 (15.8%)	3 (8.3%)		6 (14%)
Right-sided unilateral nodes	7 (36.8%)	9 (37.5%)		16 (37.2%)
Left-sided unilateral nodes	5 (26.3%)	8 (33.3%)		13 (30.2%)
Bilateral nodes	3 (15.8%)	2 (8.3%)		5 (11.6%)
Right adrenal hyperplasia	1 (5.3%)	0		1 (2.3%)
Left adrenal hyperplasia	0	2 (8.3%)		2 (4.8%)

SD, standard deviation.

**Table 2:** AVS lateralization ratios.

	Unilateral disease (*n* = 19)			Bilateral (*n* = 15)			“Grey zone” (*n* = 3)		
	Before IPCM (*n* = 5)	After IPCM (*n* = 9)	*p*	Before IPCM (*n* = 2)	After IPCM (*n* = 13)	*p*	Before IPCM (*n* = 0)	After IPCM (*n* = 3)	*p*
Median lateralization ratio (IQR)	24.1 (12.7–38)	20.8 (12.1–25.9)	0.01	1	2 (1.3–2.4)	0.1	—	3.5 (3.1-.)	—

IVC, inferior vena cava; LAV, left adrenal vein; RAV, right adrenal vein.

**Table 3:** AVS success considering preprocedural imaging.

AVS	Imaging	Bilateral		Unilateral <1 cm		Unilateral ≥1 cm		Without nodules	
		Pre-IPCM	Post-IPCM	Pre-IPCM	Post-IPCM	Pre-IPCM	Post-IPCM	Pre-IPCM	Post-IPCM
Selectivity	Yes	1	3	0	5	4	15	2	4
	No	2	1	1	0	9	0	12	0

AVS, adrenal vein sampling; IPCM, intraprocedural cortisol measurement.

## Data Availability

The clinical data of the population used to support the findings of this study are available from the corresponding author upon request.
